# Crystal structure of an oxidized mutant of human mitochondrial branched-chain aminotransferase

**DOI:** 10.1107/S2053230X19016480

**Published:** 2020-01-01

**Authors:** Darius Herbert, Synphane Gibbs, Alexys Riddick, Myra Conway, Ming Dong

**Affiliations:** a Department of Chemistry, North Carolina Agricultural and Technical State University, USA; b Department of Biology, North Carolina Agricultural and Technical State University, USA; c Department of Applied Science, University of the West of England, England

**Keywords:** human mitochondrial branched-chain aminotransferase, redox regulation, C*XX*C center, N-terminal loop, interdomain loop, transaminases

## Abstract

This study presents the crystal structure of a thiol variant of the human mitochondrial branched-chain aminotransferase protein. Human branched-chain aminotransferase (hBCAT) catalyzes the transamination of the branched-chain amino acids leucine, valine and isoleucine and α-ketoglutarate to their respective α-keto acids and glutamate. hBCAT activity is regulated by a C*XX*C center located approximately 10 Å from the active site. This redox-active center facilitates recycling between the reduced and oxidized states, representing hBCAT in its active and inactive forms, respectively. Site-directed mutagenesis of the redox sensor (Cys315) results in a significant loss of activity, with no loss of activity reported on the mutation of the resolving cysteine (Cys318), which allows the reversible formation of a disulfide bond between Cys315 and Cys318. The crystal structure of the oxidized form of the C318A variant was used to better understand the contributions of the individual cysteines and their oxidation states. The structure reveals the modified C*XX*C center in a conformation similar to that in the oxidized wild type, supporting the notion that its regulatory mechanism depends on switching the Cys315 side chain between active and inactive conformations. Moreover, the structure reveals conformational differences in the N-terminal and inter-domain region that may correlate with the inactivated state of the C*XX*C center.

## Abstract 2

The crystal structure of the oxidized form of a human mitochondrial branched-chain aminotransferase (hBCATm) mutant was determined. The structural analysis supports the concept that a complex regulation mechanism is involved in hBCATm activity, which depends on the key residue Cys315.

## 1. Introduction   

Human branched-chain aminotransferase (hBCAT) is an enzyme that catalyzes the transamination of the branched-chain amino acids leucine, valine and isoleucine and α-ketoglutarate to their respective α-keto acids and glutamate (Hall *et al.*, 1993 ▸; Hutson, 1988 ▸). These enzymes play significant roles in amino-acid metabolism and whole-body nitrogen shuttling, in particular with respect to the *de novo* synthesis of the neurotransmitter glutamate in the brain (Cole *et al.*, 2012 ▸). The hBCAT proteins are PLP-dependent enzymes and their reaction is accompanied by the interconversion of the cofactor pyridoxal 5′-phosphate (PLP) and pyridoxamine 5′-phosphate (PMP) (Birolo *et al.*, 1995 ▸; Yano *et al.*, 1992 ▸; Jäger *et al.*, 1994 ▸; Smith *et al.*, 1989 ▸). Subsequent oxidation of the resulting α-keto acids by the branched-chain α-keto acid dehydro­genase complex (BCKDC) generates acyl-CoA, which enters the TCA cycle (Brosnan & Brosnan, 2006 ▸). Mitochondrial hBCAT (hBCATm), which is found in the pancreas, kidney, stomach and brain, is thought to be responsible for the majority of transamination outside the central nervous system (Suryawan *et al.*, 1998 ▸). Meanwhile, the cytosolic hBCAT isoform (hBCATc) is predominantly expressed in the brain and the peripheral nervous system, as well as the placenta and ovary (Hall *et al.*, 1993 ▸).

Structurally, PLP-dependent enzymes have been classified into five distinct fold types (fold types I–V; Christen *et al.*, 1994 ▸; Jensen & Gu, 1996 ▸). hBCAT is a fold type IV protein (Conway *et al.*, 2002 ▸; Davoodi *et al.*, 1998 ▸; Hutson *et al.*, 1998 ▸). It forms a homodimer, and the monomer of hBCAT is composed of a small domain (residues 1–170) and a large domain (residues 182–365) connected by an interdomain loop that contains 11 amino acids (Yennawar *et al.*, 2002 ▸). The active site contains PLP, which is linked by a Schiff base to Lys202 of each monomer. The hBCAT C*XX*C center, which is unique among aminotransferases (Conway *et al.*, 2002 ▸), lies adjacent to the active site and can be modified via oxidation, including *S*-glutathionylation or *S*-nitrosylation (Conway *et al.*, 2002 ▸, 2004 ▸, 2008 ▸; Coles *et al.*, 2009 ▸). The reactive thiols of the C*XX*C center of hBCATm, Cys315 and Cys318, can form a reversible disulfide bond in response to changes in the redox environment. Structural and kinetic studies have shown that Cys315 is the ‘redox sensor’, while Cys318 allows reversible disulfide-bond formation (Conway *et al.*, 2004 ▸). The function of this redox switch has in part been characterized for hBCATm. For instance, oxidation of hBCATm regulates its ability to form a ‘metabolon’ complex with the E1 subunit of the BCKDC (Islam *et al.*, 2007 ▸) and glutamate dehydrogenase (GDH) (Islam *et al.*, 2010 ▸), enzymes that are important for the complete oxidation of the branched-chain amino acids. When oxidized, hBCATm no longer catalyzes transamination, preventing metabolite channeling through both its lack of activity and its decreased ability to stabilize multi-enzyme complexes (Islam *et al.*, 2010 ▸). Other than the C*XX*C center, a few other regions within the structure of hBCAT were identified to be important for hBCAT catalysis and include the interdomain loop (residues 171–181) that influences substrate binding and the N-terminal loop (residues 15–32), disordering of which disrupts the integrity of the substrate side-chain-binding pocket (Yennawar *et al.*, 2006 ▸).

Previous structural and kinetic analysis showed that the C*XX*C center influences hBCAT activity and was based on the analysis of C*XX*C center mutants in which no major structural changes were observed (Yennawar *et al.*, 2006 ▸). Mutation of the reactive cysteine group resulted in the largest effect on the steady-state kinetics and a subsequent loss of peroxide sensitivity (Conway *et al.*, 2004 ▸). On the other hand, re­orientation of the Cys315 side chain in the structure of the C318A mutant did not affect the activity of hBCATm (Yennawar *et al.*, 2006 ▸). However, when the C318A mutant was oxidized by H_2_O_2_ to become a C318A/C315CSD mutant the transaminase activity was almost abolished, and this loss was irreversible, representing overoxidation of the redox sensor to sulfinic/sulfonic acid (Conway *et al.*, 2004 ▸). In this study, we determined the crystal structure of the oxidized C318A mutant (C318A/C315CSD). With the goal of finding structural differences that contribute to the observed differences in activity, we compared the structure of the C318A/C315CSD mutant with that of the oxidized form of wild-type hBCATm, which has an abolished activity, and with that of the reduced form of the C318A mutant of hBCATm, which has an almost unchanged activity.

## 2. Materials and methods   

### 2.1. Macromolecule production   

The expression and purification of the C318A mutant of hBCATm was carried out as described previously (Davoodi *et al.*, 1998 ▸). In brief, the hBCATm cDNA clone was ligated into the pET-28a expression vector (kanamycin-resistant) with an N-terminal His tag and was subsequently used to transform *Escherichia coli* BL21(DE3) cells. The cells were grown in LB broth (containing 50 µ*M* kanamycin) at 310 K for 16 h and expression was induced using 1 m*M* isopropyl β-d-1-thio­galactopyranoside (IPTG) at 303 K. After 4 h, 3.7 l of cells were harvested by centrifugation. After sonication, the protein was purified using Ni–NTA resin. The final purification of hBCATm was performed using anion-exchange chromatography (Macro-Prep High Q, Bio-Rad). The final purified hBCATm protein was then dialyzed at 277 K into a buffer consisting of 25 m*M* Tris–HCl pH 7.5, 150 m*M* NaCl, 1 m*M* glucose, 1 m*M* EDTA, 1 m*M* α-ketoisocaproate, 5 m*M* DTT, 15% glycerol at pH 7.5. The protein was flash-cooled and stored at 193 K. The concentration of the purified protein was estimated using the absorbance at 280 nm with an extinction coefficient of 67 600 *M* ^−1^ cm^−1^ per monomer. Macromolecule-production information is summarized in Table 1 ▸.

### 2.2. Crystallization   

The C318A mutant was subjected to sparse-matrix crystallization screening using the hanging-drop vapor-diffusion method (McPherson, 1982 ▸). Screening kits from Hampton Research were used for screening. Needle-shaped crystals of the C318A mutant were grown (Table 2 ▸). In order to determine the structure of the oxidized C318A mutant, the protein crystals obtained were incubated in crystallization buffer with the addition of 1% hydrogen peroxide before applying the cryoprotectant.

### 2.3. Data collection and processing   

The crystals were cryoprotected by transferring them into perfluoropolyether cryo oil. X-ray diffraction data were collected at the Wake Forest School of Medicine X-ray facility (Table 3 ▸). The diffraction data were indexed, integrated and scaled using *HKL*-3000 (Minor *et al.*, 2006 ▸). The data were truncated at 3.3 Å resolution to maintain completeness.

### 2.4. Structure solution and refinement   

*MOLREP* from *CCP*4 (Winn *et al.*, 2011 ▸) was used for molecular replacement using the structure of the C318A mutant (PDB entry 2hgw; Yennawar *et al.*, 2006 ▸) as a search model. The model was built and the thiol at Cys315 was replaced with sulfinic acid. The coordinates were refined using *REFMAC*5 from *CCP*4 and *Coot* (Emsley *et al.*, 2010 ▸). The final model was composed of PLP, residues 3–23, 27–171 and 178–365 of one monomer, residues 3–22 and 28–365 of a second monomer and no water molecules. The structure was validated using *MolProbity* within the *Phenix* suite (Liebschner *et al.*, 2019 ▸). *UCSF Chimera* was used to present the structures (Pettersen *et al.*, 2004 ▸). Refinement statistics are summarized in Table 4 ▸.

## 3. Results and discussion   

**Figure 1:**
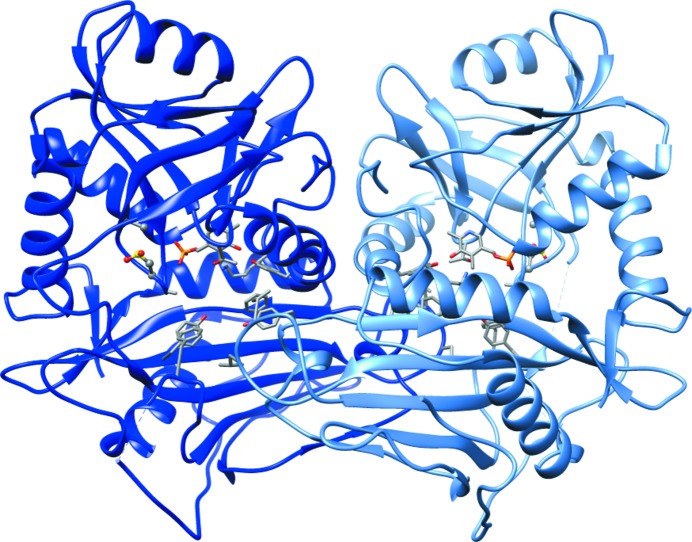
The overall structure of the C318A/C315CSD mutant of hBCATm (cartoon; chain *A*, blue; chain *B*, light blue) resembles that of wild-type hBCATm. The structure is a homodimer with the PLP linked to Lys202 by a Schiff base. The active-site residues and the C*XX*C center are shown in stick representation. The phosphate of PLP is shown in orange. (Atom color scheme: carbon, gray; nitrogen, blue; oxygen, red; phosphate, orange; sulfur, yellow.)

**Figure 2:**
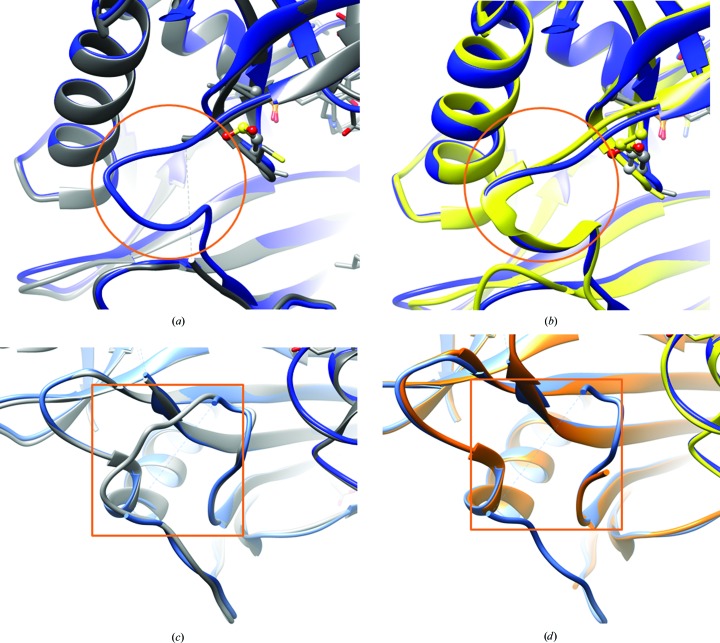
(*a*, *b*) Close-up of an inter-domain loop overlay of the crystal structures of (*a*) the C318A (gray; PDB entry 2hgw; Yennawar *et al.*, 2006 ▸) and C318A/C315CSD (blue) mutants and (*b*) the oxidized wild type (orange/yellow; PDB entry 2hhf; Yennawar *et al.*, 2006 ▸) and the C318A/C315CSD mutant (blue). The interdomain loop (residues 171–181, highlighted by the circle), which was missing in the C318A mutant owing to a lack of electron density, was interpreted in one of the monomers of the C318A/C315CSD mutant (*a*) and mimics the interdomain loop of oxidized wild-type hBCATm (*b*). (*c*, *d*) Close-up of an N-terminal loop overlay of the crystal structures of (*c*) the C318A (gray; PDB entry 2hgw) and C318A/C315CSD (blue) mutants and (*d*) the oxidized wild type (orange/yellow; PDB entry 2hhf) and the C318A/C315CSD mutant (blue). The N-terminal loop (residues 15–32, highlighted by the square) of the C318A mutant is interpreted in both monomers, whereas it is much more flexible and remains uninterpreted in both monomers of the C318A/C315CSD mutant (*c*) and one monomer of the oxidized wild type (*d*).

We determined the structure of the C318A/C315CSD variant of hBCATm (Fig. 1 ▸). The overall structure resembles the canonical hBCATm homodimer and contains a Schiff base-linked PLP in the active sites, which are located at the interface between the small and large domains of each monomer. The overall structure of the C318A/C315CSD variant overlays very well with that of the C318A mutant and the oxidized form of wild-type hBCATm (Supplementary Fig. S1), with a few exceptions in the interdomain loop (residues 171–181) and the N-terminal loop (residues 15–32) (Fig. 2 ▸). The interdomain loop, which was not interpretable in the structure of the C318A mutant owing to a lack of electron density, is interpreted in one of the monomers of the C318A/C315CSD structure, which overlays very well with the interdomain loop of the oxidized form of wild-type hBCAT. On the other hand, the N-terminal loop was not interpreted in either monomer of the C318A/C315CSD mutant, while the N-terminal loop was interpreted in only one of the monomers of the oxidized form of wild-type hBCATm and was interpreted in both monomers of the C318A mutant.

**Figure 3:**
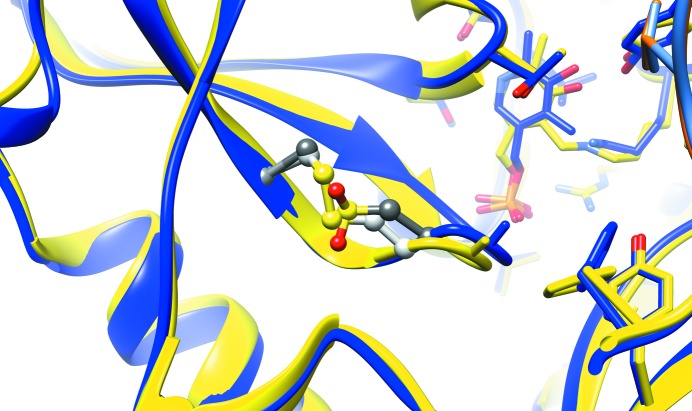
Close-up of the overlay of the C*XX*C center of the crystal structures of oxidized wild-type hBCATm (yellow ribbon with Cys315 and Cys318 in light gray) and the C318A/C315CSD mutant (blue ribbon with C315CSD and C318A in dark gray). The orientation of C315CSD resembles that of Cys315 in the disulfide-bonded form. (Atom color scheme: carbon, gray and light gray; oxygen, red; sulfur, yellow.)

In the structure, C318A and C315CSD follow the β-turn (residues 311–314) adjacent to the active site. Conformationally, they resemble the C*XX*C center of oxidized wild-type hBCATm (Fig. 3 ▸), with the oxidized C315CSD having a similar orientation to the disulfide bridge in the oxidized wild type. The substrate-binding pocket includes the residues Phe30, Yal173 and Val155 which interact with the substrate when first entering the active site, and Tyr207, Phe75, Tyr141, Thr240, Ala314, Val155, Tyr70 and Leu153 which form the hydrophobic pocket of the side-chain-binding pocket (Yennawar *et al.*, 2006 ▸). Most of the residues overlay well with the oxidized form of wild-type hBCATm, except for Tyr173, which was not interpreted in one monomer owing to an apparently dis­ordered interdomain loop. These observations are consistent with previous structural analysis that suggested that the C315A mutation could lead to a loss of its original interaction with Tyr173 via an S–H π bond (Yennawar *et al.*, 2006 ▸), which in turn could result in a greater flexibility of Tyr173.

Overall, in comparison to the known wild-type and mutant structures, the structural features of the C318A/C315CSD mutant correlate closely with its kinetic parameters. These correlations involve the conformation of the C*XX*C center, the interdomain loop and the N-terminal loop. For the C*XX*C center (Yennawar *et al.*, 2006 ▸), it has previously been indicated that the dipole formed by Cys315 and Gly312 contributes to stabilizing the phosphate of the PLP ligand and the carboxylate of the incoming substrate. Therefore, a stronger β-turn dipole in the C318A/C315CSD mutant could impede the substrate orientation similarly to that previously described for the Cys315 mutant (Yennawar *et al.*, 2006 ▸). On top of this, the structure of the C318A/C315CSD mutant reveals a modified C*XX*C center and also one interdomain loop in a conformation similar to the oxidized wild type. The interdomain loop is known to affect substrate binding via modulating access to the active site (Conway *et al.*, 2003 ▸). The interpreted interdomain loop in one C318A/C315CSD monomer resembles that in the oxidized form of wild-type hBCATm, coinciding with their similar diminished activities. Lastly, we observe a systematic correlation concerning the disordering of the N-terminal loop, which could disrupt the integrity of the side-chain-binding pocket of the substrate (Yennawar *et al.*, 2006 ▸). Compared with the reduced wild type, the N-terminal loop of the C318A mutant showed the fewest changes, while both the oxidized wild type and the C318A/C315CSD mutant showed very flexible and uninterpreted and apparently flexible N-terminal loops, echoing the observed differences in kinetic properties between these constructs. This observation is consistent with previous structural and kinetics analyses of the C315A and C318A mutants, in which the differences in activity were postulated to be contributed by flexibility of the N-terminal loop (Yennawar *et al.*, 2006 ▸). Overall, our structural analysis supports the concept that a complex regulation mechanism is involved in hBCATm activity, which depends on the key residue Cys315 (Yennawar *et al.*, 2006 ▸; Conway *et al.*, 2002 ▸, 2004 ▸). 

## Supplementary Material

PDB reference: human mitochondrial branched-chain aminotransferase, 6prx

supplement figures. DOI: 10.1107/S2053230X19016480/rf5027sup1.pdf

## Figures and Tables

**Table 1:** Macromolecule-production information

Source organism	*Homo sapiens*
Expression vector	pET-28a
Expression host	*E. coli*
Complete amino-acid sequence of the construct produced	MGGSHHHHHHGMASGSHMASSSFKAADLQLEMTQKPHKKPGPGEPLVFGKTFTDHMLMVEWNDKGWGQPRIQPFQNLTLHPASSSLHYSLQLFEGMKAFKGKDQQVRLFRPWLNMDRMLRSAMRLCLPSFDKLELLECIRRLIEVDKDWVPDAAGTSLYVRPVLIGNEPSLGVSQPRRALLFVILCPVGAYFPGGSVTPVSLLADPAFIRAWVGGVGNYKLGGNYGPTVLVQQEALKRGCEQVLWLYGPDHQLTEVGTMNIFVYWTHEDGVLELVTPPLNGVILPGVVRQSLLDMAQTWGEFRVVERTITMKQLLRALEEGRVREVFGSGTACQVAPVHRILYKDRNLHIPTMENGPELILRFQKELKEIQYGIRAHEWMFPV

**Table 2:** Crystallization

Method	Vapor diffusion
Plate type	Hanging drop
Temperature (K)	293
Protein concentration (mg ml^−1^)	5
Buffer composition of protein solution	Potassium phosphate pH 7.5
Composition of reservoir solution	200 m*M* magnesium acetate tetrahydrate, 100 m*M* sodium cacodylate trihydrate pH 6.5, 20%(*w*/*v*) polyethylene glycol 8000
Volume and ratio of drop	1:1
Volume of reservoir (µl)	500

**Table 3:** Data collection and processing Values in parentheses are for the outer shell.

Diffraction source	Copper rotating anode
Wavelength (Å)	1.54180
Temperature (K)	100
Detector	PILATUS3 R 200K-A
Crystal-to-detector distance (mm)	90
Rotation range per image (°)	0.5
Exposure time per image (s)	150
Space group	*P*2_1_2_1_2_1_
*a*, *b*, *c* (Å)	69.20, 104.94, 106.29
α, β, γ (°)	90, 90, 90
Mosaicity (°)	1.0–1.5
Resolution range (Å)	50–3.22 (3.28–3.22)
CC_1/2_	0.871 (0.879)
No. of unique reflections	12545 (586)
Completeness (%)	99.1 (92.9)
Multiplicity	8.8 (2.1)
〈*I*/σ(*I*)〉	11.9 (2.22)
*R*_r.i.m._	0.04 (0.23)
Overall *B* factor from Wilson plot (Å^2^)	44

**Table 4:** Structure refinement Values in parentheses are for the outer shell.

Resolution range (Å)	47.46–3.25 (3.28–3.25)
Completeness (%)	98.5 (92.9)
σ Cutoff	*F*/σ > 1.36
No. of reflections, working set	12739 (780)
No. of reflections, test set	602 (32)
Final *R*_cryst_	0.22 (0.30)
Final *R*_free_	0.259 (0.33)
Estimated coordinate error	0.43
No. of non-H atoms	
Protein	5615
Ligand	30
Water	0
Total	5645
R.m.s. deviations	
Bonds (Å)	0.019
Angles (°)	1.1
Average *B* factors (Å^2^)	44
Protein	31
Ligand	36
Ramachandran plot	
Favored regions (%)	91.9
Additionally allowed (%)	7.1
Outliers (%)	0.9
