# Supplementary material for: Crystal structure of an oxidized mutant of human mitochondrial branched-chain aminotransferase
Source: Acta Crystallogr F Struct Biol Commun. 2020 Jan 1;76(Pt 1):14–9. doi: 10.1107/S2053230X19016480 (PMC6957111; doi:10.1107/S2053230X19016480)
Supplement: Supplementary file 1 [file f-76-00014-sup1.pdf]

STRUCTURAL BIOLOGY
COMMUNICATIONS

Volume 76 (2020)

Supporting information for article:

Crystal structure of an oxidized mutant of human mitochondrial branched-chain aminotransferase

Darius Herbert, Synphane Gibbs, Alexys Riddick, Myra Conway and Ming Dong

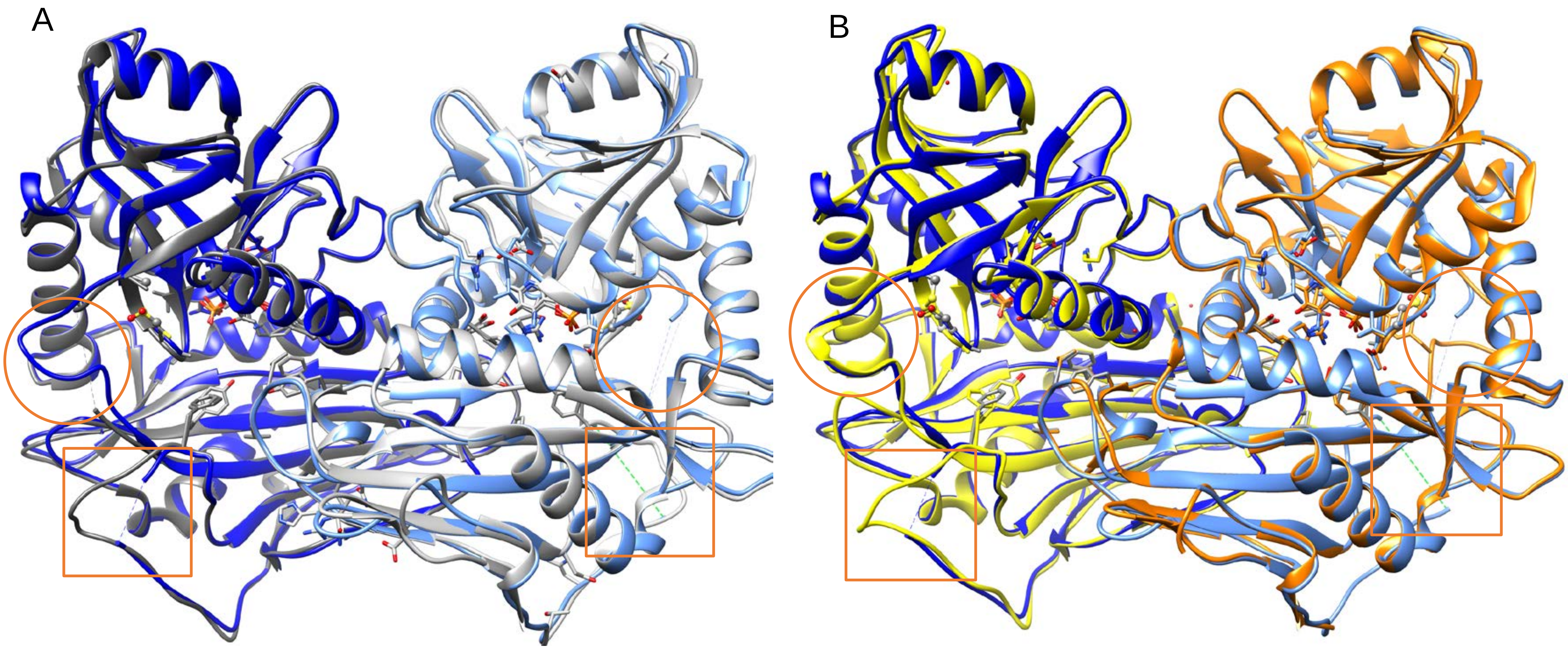

Supplementary Figure S1. A. Overlay of the crystal structures of C318A (grey) (pdb:2hgw) and C318A/C315CSD (blue). The overall structures superimpose very well, with a few exceptions of two regions. The N-term loop (15-32, in square) of C318A is interpreted in both monomers, whereas much more apparently flexible and un-interpreted in both monomers of the C318A/C315CSD. The interdomain loops (171-181, in circle) which were missing in C318A due lack of electron density were interpreted in one of the monomers of the C318A/C315CSD. **B.** Overlay of the crystal structures of oxidized wild type (orange/ yellow) (pdb:2hhf) and C318A/C315CSD (blue). The overall structures superimpose very well with similar flexibility in their N-term loops. The N-term loop (15-32, in square) which is un-interpreted in both C318A/C315CSD monomers, is also missing in one monomer of the oxidized wild type (in square of the right monomer). Moreover, the interdomain loop of one C318A/C315CSD monomer is fully ordered in the same conformation as in the oxidized wild type (in circle of the left monomer).

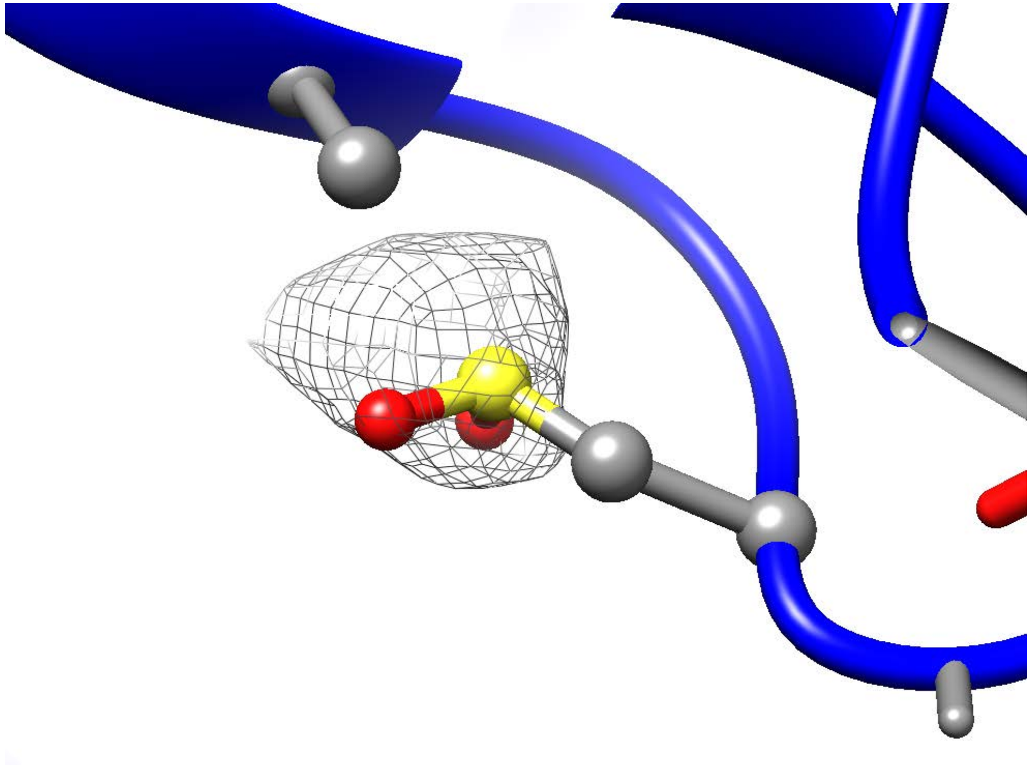

Supplementary Figure S2. CSD315 is shown with an omit map contoured at 4.0 σ. The chain A of the C318A/C315CSD mutant of hBCATm is shown in blue. The active site residues and the CXXC center are shown in stick representation. (Color scheme of atoms: as in Figure 1)
